# Everybody's Page

**Published:** 1888-12-08

**Authors:** 


### Everybody's Page.

A child's arm was recently found on the beach at Clifton, Rhode Island, and was duly sat upon by a coroner and his twelve good men and true. After an hour's deliberation they brought in a verdict that "the arm came to its death from diseases unknown to the jury." They were prudent Yankees, and did not commit themselves to any opinion as to the fate of the body originally appended to the arm in question.

Cases are not unknown where physical pain has driven sufferers to suicide; but very few of these show such a lack of perception of possibilities as a certain James Daly, who, being maddened with sciatica, tried to drown himself in a pool on Putney Heath. Fortunately he was rescued before death resulted, and his punishment for his folly was severe enough, for the immersion in cold water greatly increased the severity of his already agonising complaint.

It has been noticed that dwellers in deep valleys, where the light of the sun never strongly penetrates, are often stunted in body and weak in mind. The gloom of the mountains overshadows them, and infects with sadness their literature, their music, even their superstitions. They have on the whole more depth of feeling than dwellers on plains, but they are not so cheerful, for sunshine is essential to happiness.

A medical man, wishing to gain a few statistics regarding the dangers attending athletics, sent a list of questions on the subject to a number of schoolmasters. Most of these are of opinion that the dangers attendant on cricket and football are greatly exaggerated; but several of them add a rider to the effect that they think the spectators of such sport are in considerable danger. "I consider," says one, "that the looking on at football and athletic sports is our most fertile source of coughs and colds, and the illnesses following upon them."

Everybody knows that the thumb is a very important organ. Its Latin name, *pollex*, is derived from a word which signifies power, and we use the phrase, "I have got him under my thumb," to imply the supreme control which one man may have over another. But the Esquimaux go so far as to believe that woman was created from the thumb of man. Does this myth imply an Esquimaux notion that man should be in subjection to woman, that he is, so to speak, under his own thumb.

In the recently-published "Letters of Charles Kirkpatrick Sharpe" an anecdote is told of an impecunious Scottish baronet who made one of his sons an apothecary at Carlisle, and said, as he parted with him, "God speed ye! Ye'll revenge the fecht at Flodden." The Scotch certainly have long memories for national grievances, but it is to be hoped that this patriotic parent was a unique specimen. It is some time since Flodden was fought, and we don't like to think of the diplomas of Edinburgh, Glasgow, and Aberdeen as weapons of offence.

Once upon a time there were some bold Englishmen who started a cricket club at Riga, in Russia. The authorities heard of this, and evolved from their red-tape-bound consciousness the idea that the game was dangerous, so they sent a policeman to observe and report. Now the policeman was conscientious, and being desirous to see the game as thoroughly as possible he insisted on standing among the players. The result of this was that he joined in it unconsciously—that is, he fielded a ball with his head, and his skull being rather softer than a swift-flying cricket ball, he was more or less injured. This convinced the wise authorities that their views as to the dangers of cricket were well founded, and the game was forbidden by order of the municipality.

Overheated rooms are a great mistake. They tend to make those who sit in them unduly sensitive to cold. We are sufficiently guilty in this matter, and sacrifice health to what we call comfort, but our American cousins are worse; and it is possible that the indoor atmosphere produced by their hot stoves has much to do with the avowed increase of pneumonia and other signs of physical deterioration.

Some years ago a Royal Commission was bold enough to advise that candidates for army appointments should be examined in physical accomplishments—rowing, gymnastics, running, leaping—and get a moderate number of marks for them—not many marks, but enough to bring about a race of officers who would be only able-bodied idiots. The suggestion was rejected, and merely intellectual tests were chosen. Yet surely physique counts for something in a soldier's efficacy. There are many men fit to lead a forlorn hope, and make it something less than forlorn, who could not pass the *pons asinorum*.

The Legislature of the State of Illinois has passed a law making the intermarriage of cousins illegal. The Legislature of the State of Illinois is a rash and presumptuous pseudo-scientific body. The proofs that evil results follow from marriages of consanguinity are by no means so convincing as to justify such a high-handed law. The marriage of cousins of different temperaments—and in love the rule is not that like draws to like, but the opposite—especially if they have been brought up under different physical conditions, is by no means such a dangerous matter as the legislators of Illinois seem to think.

Even people who are in the habit of taking alcohol as part of their daily food abstain from it when any exertion demanding special accuracy is required of them. One mighty Nimrod is said to declare that a single glass of sherry with his lunch spoils his shooting for the day, and takes a flask of cold tea with him to the moors; while a famous violinist, who is subject, as men of genius often are, to fits of nervousness when about to appear before an audience, refuses to give himself "Dutch courage" by a single glass of wine. He says it would spoil his playing; he would blurr the notes if he took it.

The enervating effect of a hot bath has brought upon it clerical as well as medical disapproval, and at one time it was absolutely placed under the ban of the Church. That, however, was in the bygone days, when cleanliness was looked upon as inconsistent with any special degree of piety; and, though the hot bath is not to be recommended to anyone with a tendency to faint, it is useful for taking away the feeling of stiffness that usually follows any unusual exertion. Napoleon used to indulge in a hot bath after a fatiguing day, and found it more refreshing than even sleep. In fact, it enabled him to dispense with sleep, and so gave him a great advantage over rival generals.

It is almost a hopeless doctrine to preach that persons who inherit a tendency to consumption should not marry. Human nature is wilful, and love will still be lord of all. But a great deal might be done to prevent the transmission of the disease if marriage were postponed till after the thirtieth year was past, as phthisis is most apt to appear between the ages of twenty and thirty. Nervous children are, in many cases, better brought up away from home. It is highly proable that they inherit their delicacy from parents of similar constitution, and the rule of the neurotic parent over the neurotic child is apt to be so capricious—now foolishly lax and again needlessly severe—that it is apt to develop a nervous tendency into actual disease.

